# A novel method for the quantitative assessment of the fitted containment efficiency of face coverings

**DOI:** 10.1017/ice.2022.316

**Published:** 2023-09

**Authors:** William D. Bennett, Steven E. Prince, Kirby L. Zeman, Hao Chen, James M. Samet

**Affiliations:** 1 University of North Carolina at Chapel Hill, Chapel Hill, North Carolina; 2 Center for Public Health and Environmental Assessment, US Environmental Protection Agency, Research Triangle Park, North Carolina; 3 Oak Ridge Institute for Science Education, Oak Ridge, Tennessee

## Abstract

**Background:**

Face masks reduce disease transmission by protecting the wearer from inhaled pathogens and reducing the emission of infectious aerosols. Although methods quantifying efficiency for wearer protection are established, current methods for assessing face mask containment efficiency rely on measurement of a low concentration of aerosols emitted from an infected or noninfected individual.

**Methods:**

A small port enabled the introduction of 0.05 µm sodium chloride particles at a constant rate behind the mask worn by a study participant. A condensation particle counter monitored ambient particle numbers 60 cm in front of the participant over 3-minute periods of rest, speaking, and coughing. The containment efficiency (%) for each mask and procedure was calculated as follows: 100 × (1 − average ambient concentration with face covering worn/average ambient concentration with a sham face covering in place). The protection efficiency (%) was also measured using previously published methods. The probability of transmission (%) from infected to uninfected (a function of both the containment efficiency and the protection efficiency) was calculated as follows: {1 − (containment efficiency/100)}×{1 − (protection efficiency/100)}×100.

**Results:**

The average containment efficiencies for each mask over all procedures and repeated measures were 94.6%, 60.9%, 38.8%, and 43.2%, respectively, for the N95 mask, the KN95 mask, the procedure face mask, and the gaiter. The corresponding protection efficiencies for each mask were 99.0%, 63.7%, 45.3%, and 24.2%, respectively. For example, the transmission probability for 1 infected and 1 uninfected individual in close proximity was ∼14.2% for KN95 masks, compared to 36%–39% when only 1 individual wore a KN95 mask.

**Conclusion:**

Overall, we detected a good correlation between the protection and containment that a face covering afforded to a wearer.

A principal tool for reducing the transmission of severe acute respiratory coronavirus virus 2 (SARS-CoV-2), face masks protect the wearer from inhaled aerosol-laden virus in the environment and reduce the concentration of aerosols that infected individuals release into the environment. Fitted filtration methods that quantify protection efficiency for the wearer have been well established.^1–4^ By contrast, current methods for assessing the effectiveness of face coverings for source control are generally semiquantitative and rely on the measurement of very low concentrations of aerosols emitted from a healthy or infected individual.^5–8^ Alternatively, such measures involve the use of mannequins^9^ in which a high concentration of surrogate aerosols can be introduced within the mask. Quantitative measurement of the containment efficiency of face coverings as fitted on a living person is lacking. In the present study, we report a novel method for the quantitative assessment of the containment efficiency for face coverings commonly worn by the public during the coronavirus disease 2019 (COVID-19) pandemic. We used this method to determine the correlation between the protection provided to the wearer (protection efficiency) and that afforded to others from the wearer (containment efficiency).

## Methods

**Fig. 1.**
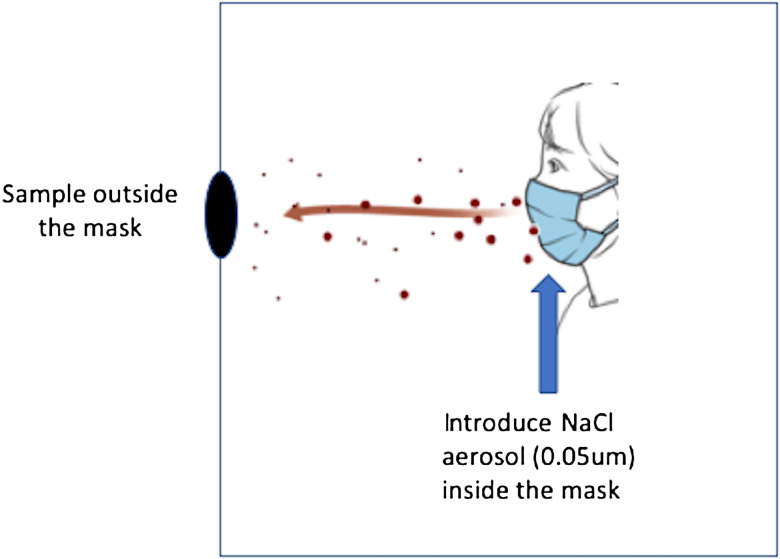
Schematic of ventilation chamber to accommodate a seated study participant, introduce a fixed aerosol concentration behind the mask, and measure ambient concentration that escapes from inside the mask.

Expanding on our standard methods used for fitted protection efficiency,^1–3^ we designed a small-volume (0.8 m^3^), low-ventilation chamber to accommodate a seated study participant, an adult male (weight, 75 kg; height, 178 cm; head size, 58.5 cm; and no beard) (Fig. 1). The participant wore 5 different face coverings: (1) an N95 mask (model number 9210, 3M, Maplewood, MN); (2) KN95 (Lei Shi De, EN149-2001+A1:2009, CIRS Garments, Shandong, PRC); (3) a nonmedical-grade procedure mask (Blue Mask, Jiangsu Jianyu Health Medical Company, Jiangsu, PRC); (4) a gaiter (92% polyester, 8% spandex, TICONN US, Stony Brook, NY); or (5) a sham face covering (N95 described above with head straps and outer frame remaining, and all other material cut out, with the exception of a <1-cm thin center strip that allowed for insertion of a metal port). Each face covering had a port in the center to allow introduction of a stream of nebulized 0.05 μm sodium chloride particles (TSI 8026 Particle Generator, TSI, Shoreview, MN) at a constant rate into the mask space. The particles had a count median diameter (CMD) of ∼0.05 μm, as measured by a scanning mobility particle sizer. The ambient chamber concentration was continuously measured using a TSI 3775 condensation particle counter (CPC), which took samples 60 cm in front of the participant’s head over a series of three 3-minute periods while the participant engaged in (1) resting breathing, (2) reading out loud (rainbow passage), and (3) coughing forcefully (2×10 coughs, ∼450 L/min peak flows). The coughing maneuver was included to represent the most extreme impact on containment relative to resting breathing or reading. Particle size was essentially unchanged in the ambient space following emission (∼CMD, 0.05 μm). The fitted containment efficiency (% CE) for each mask or procedure was calculated as follows:
(Eq. 1)

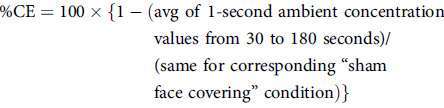




The protection efficiency (%PE) was also measured in the same chamber using a previously described modified National Institute for Occupational Safety and Health (NIOSH) fit-testing method^1–3^ using the same conditions as the test for containment efficiency (ie, resting breathing, reading, and coughing). To bolster reliability, data collection consisted of 3 repeated measures of containment efficiency and protection efficiency for each mask. Using the mean of the 3 repeated measures of containment efficiency and protection efficiency for a given combination of masks worn by infected and uninfected individuals, we calculated the theoretical probability of transmission as follows:
(Eq. 2)

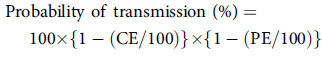




Notably, this index of transmission only reflects the fitted filtration for protection and containment of the masks tested and does not account for other factors known to influence viral transmission (eg, viral loads, symptom presence, time and space exposures, etc).

The institutional review board at the University of North Carolina at Chapel Hill waived the need for approval of these studies as well as the requirement to obtain individual consent for device testing.

## Results

**Fig. 2.**
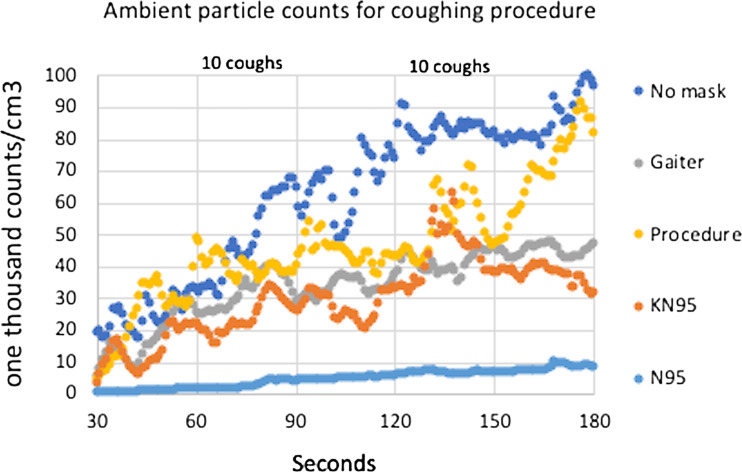
Sample plot of ambient condensation particle counter (CPC) counts over time for each mask during a coughing maneuver.

Figure 2 illustrates an example of particle counts measured over time in the chamber atmosphere as the aerosol escaped from each of 4 masks worn by the adult male study participant. Table 1 shows the mean ambient CPC counts (collected over 30–180 seconds) and calculated containment efficiency (Eq. 1) for the sample data illustrated in Figure 2. Table 2a shows the mean containment efficiency (±SD) for each mask over all maneuvers performed and repeated measures. The containment efficiency for the N95 mask was unaffected by the talking or coughing maneuvers, whereas the 2 ear-loop masks (KN95 and procedure) were relatively less efficient at containment, especially during the coughing maneuver, compared to the resting and reading maneuvers. The gaiter measurements, by contrast, showed an unexpected improvement for both the reading and coughing maneuvers. Table 2b shows the mean protection efficiency (±SD) for each mask over all maneuvers performed and repeated maneuvers. Like the containment efficiency, the protection efficiency for the N95 was unaffected by talking or coughing compared to resting breathing. The 2 ear-loop masks were less efficient at protection than the N95, especially the KN95, during reading and coughing. Like the containment efficiency, the protection efficiency showed some improvement with reading in the gaiter.

**Table 1.** Sample ambient Condensation Particle Counter (CPC) Data^a^ for a Measure of Containment Efficiency (%CE) Associated With a Single Coughing Maneuver in Each of the 4 Masks

Mask Type	Average CPC CountsOver 30–180 Seconds	%CE
No mask	62,107	
N95	4,818	92.2
KN95	29,736	52.1
Procedure	47,136	24.1
Gaiter	34,163	45.0

a Also see Figure 2.

**Table 2a.** Comparison of Containment Efficiencies (%CE) for the 3 Maneuvers

Mask Type	Mean %CE (SD)		
	Rest	Read	Cough
N95	94.5 (1.1)	94.9 (0.5)	94.5 (2.1)
KN95	64.5 (5.5)	64.5 (1.2)	53.7 (3.1)
Procedure	43.7 (22.3)	41.0 (6.2)	31.6 (6.5)
Gaiter	34.1 (14.4)	45.3 (1.0)	50.1 (5.1)

Note. SD, standard deviation.

**Table 2b.** Comparison of the Protection Efficiencies (%PE) for the 3 Maneuvers

Mask Type	Mean %PE (SD)		
	Rest	Read	Cough
N95	99.7 (0.1)	98.6 (0.2)	98.3 (0.2)
KN95	75.2 (4.2)	60.6 (6.0)	52.5 (8.2)
Procedure	45.5 (23)	53.1 (8.9)	38.7 (5.7)
Gaiter	19.6 (21.8)	31.1 (12.6)	25.3 (8.0)

Note. SD, standard deviation.

Table 3 shows the comparison between the average containment efficiency and the average protection efficiency over all maneuvers and for all masks. With the notable exception of the gaiter, containment efficiency followed the same rank order and performance level as did protection efficiency, with a trend for containment efficiency that was slightly less than protection efficiency. In contrast, the efficiency of the gaiter appeared to display better containment than protection.

**Table 3.** Comparison of Mean Containment Efficiencies (%CE) to the Protection Efficiencies (%PE) Over All Maneuvers

Mask Type	Mean %CE (SD)	Mean %PE (SD)
N95	94.6 (0.5)	99.0 (0.2)
KN95	60.9 (2.7)	63.7 (4.5)
Procedure	38.8 (7.4)	45.3 (12.8)
Gaiter	43.2 (3.5)	24.2 (16.0)

Note. SD, standard deviation.

Using the data given in Table 3, Table 4 shows calculations for theoretical transmission probabilities (Eq. 2) associated with an infected and uninfected individual wearing any (or none) of the 4 masks tested in this study. The diagonal data in bold/underline and green highlights the case of each individual wearing the same type of mask (or none) and ranges from 0.1% to 100%. Any combination of masks worn by 2 individuals, one infected and the other uninfected, results in <50% transmission probability of aerosol to the uninfected (Table 4). The worst transmission probability was 76% for the case of a gaiter worn by the uninfected and no mask for the infected.

**Table 4.**
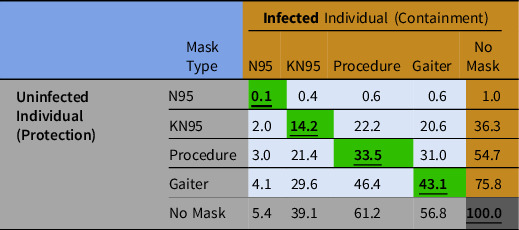
Theoretical transmission probability (%) to uninfected from infected for paired masks. Calculation is based on wear by infected individual for containment (horizontal) and wear by uninfected individual for protection (vertical). Comparison also shown for ‘no mask’ conditions in final column (infected individual) and row (uninfected individual). Diagonal transmission probabilities in bold/underlined/green highlight the case of identical mask types worn by each individual.

## Discussion

We present a novel quantitative method for the evaluation of the fitted containment efficiency of face coverings. The rank order for the containment efficiency of the 4 masks we tested closely parallels that of their protection efficiency. For both measures, we introduced coughing as a test maneuver (Fig. 1) that would represent a worst-case scenario for both containment and protection. For all masks except the gaiter, containment tended to be reduced during coughing compared to resting breathing and reading. In terms of percentage, the ear-loop masks (KN95 and procedure) tended to be less efficient for containment than for protection. We hypothesize that this finding was related to increased positive mask pressure during expiration (ie, a factor in determining collection efficiency) compared to negative pressure on inhalation (ie, a determinant of protection efficiency). On the other hand, the gaiter mask was more efficient at containing aerosols in the mask than it was at excluding them. The different containment behavior for the gaiter relative to the other masks may be due to the extension of the gaiter material down over the neck, which effectively served as a reservoir for collection of exhaled aerosol that escape in other masks.

Not surprisingly given previous findings, the 3M N95 (model 9210) mask was very efficient at both collection and protection; it essentially protected equally well against aerosol transmission when worn by either an infected or uninfected individual.^2,4^ The protection efficiency rate tested here was measured previously using the OSHA quantitative fit-testing protocol as 98.4% (SD, ±0.05) for the 3M 9210 in the same individual^2^ compared to 99% (SD, ±0.02) in the current study. Furthermore, high-efficiency respirators (ie, N95 and KN95) as worn and tested by a male study participant, provided better containment and protection efficiency rates compared to a nonmedical procedure mask and gaiter.

Our previous findings for double masking of a comparable procedure mask (Shine Ya),^3^ worn only by an uninfected study participant, showed a 45% transmission probability compared to 33% (Table 4) when both were wearing the procedure mask studied here. These data support the simple notion that optimal masking for prevention of viral transmission is captured by the situation in which both the uninfected and infected are wearing a mask.

SARS-CoV-2 virions are 50–200 nm in diameter,^10^ but they can also be transmitted in much larger droplets. The CMD of ambient particles used in this experiment was ∼50 nm, likely smaller than SARS-CoV-2 virions or droplets containing the virus. However, the particle size of the ambient aerosol was very similar to the sodium chloride particle size used to test and certify N95 respirators (75 ± 20 nm), which is deemed appropriate by the US Occupational Safety and Health Administration.^4^ Based on the mechanisms that govern particle deposition and filtration by face masks (ie, diffusion, impaction, interception, and sedimentation), it is clear that protection against aerosols with a count median diameter of 50 nm would also confer similar or better protection against much larger aerosols or droplets >3 μm.^11^ In fact, for masks with an electric charge (like those tested in this study), the most penetrating particle size was 30–60 nm,^12^ which is similar in size to those used in this study.

This study had several limitations. We tested each mask on a single adult male rather than many study participants with varying facial configurations. On the other hand, testing a single individual allowed comparison to fitted filtration efficiencies measured with the same study participant in previous tests of a larger range of masks.^1–3^ Repeated measures (n = 3) in this single participant also provided us with a measure of intrasubject variability (Tables 2 and 3, SD) for fitted-mask containment and protection.

In conclusion, masks commonly used by the public exhibit a range of containment and protection efficiencies. Overall, there was a good correlation between the fitted containment and protection performances of the masks we tested. Coughing tended to reduce mask containment for all but the gaiter mask. Modeling showed that high-quality respirators can reduce theoretical transmission when worn for either containment or protection. The theoretical probabilities in Table 4 did not consider the absolute ambient virion dose, but the probabilities can be applied to these absolute levels to determine what fraction of the ambient virions can be transmitted to a mask wearer. The estimated transmission probability for any given mask type is always lower if there are 2 mask wearers relative to only 1, indicating that the best masking practice for reducing aerosol transmission of viruses or other pathogens is realized when everyone wears a mask.
